# Cell Aggregation Capability of Clinical Isolates from *Candida auris* and *Candida haemulonii* Species Complex

**DOI:** 10.3390/tropicalmed8080382

**Published:** 2023-07-27

**Authors:** Lívia S. Ramos, Claudia M. Parra-Giraldo, Marta H. Branquinha, André L. S. Santos

**Affiliations:** 1 Laboratório de Estudos Avançados de Microrganismos Emergentes e Resistentes (LEAMER), Departamento de Microbiologia Geral, Instituto de Microbiologia Paulo de Góes (IMPG), Universidade Federal do Rio de Janeiro (UFRJ), Rio de Janeiro 21941-902, Brazil; liviaramos2@yahoo.com.br (L.S.R.); mbranquinha@micro.ufrj.br (M.H.B.); 2 Unidad de Proteómica y Micosis Humanas, Grupo de Enfermedades Infecciosas, Departamento de Microbiología, Facultad de Ciencias, Pontificia Universidad Javeriana, Bogotá 110231, Colombia; claudia.parra@javeriana.edu.co; 3 Rede Micologia RJ—Fundação de Amparo à Pesquisa do Estado do Rio de Janeiro (FAPERJ), Rio de Janeiro 21941-902, Brazil; 4 Programa de Pós-Graduação em Bioquímica, Instituto de Química, Universidade Federal do Rio de Janeiro (UFRJ), Rio de Janeiro 21941-909, Brazil

**Keywords:** *Candida haemulonii* clade, cell-cell interaction, emerging yeasts, drug resistance, virulence, physicochemical conditions

## Abstract

The opportunistic fungal pathogens belonging to the *Candida haemulonii* complex and the phylogenetically related species *Candida auris* are well-known for causing infections that are difficult to treat due to their multidrug-resistance profiles. *Candida auris* is even more worrisome due to its ability to cause outbreaks in healthcare settings. These emerging yeasts produce a wide range of virulence factors that facilitate the development of the infectious process. In recent years, the aggregative phenotype has been receiving attention, as it is mainly associated with defects in cellular division and its possible involvement in helping the fungus to escape from the host immune responses. In the present study, we initially investigated the aggregation ability of 18 clinical isolates belonging to the *C. haemulonii* species complex (*C. haemulonii sensu stricto*, *C. duobushaemulonii*, and *C. haemulonii* var. *vulnera*) and *C. auris*. Subsequently, we evaluated the effects of physicochemical factors on fungal aggregation competence. The results demonstrated that cell-to-cell aggregation was a typically time-dependent event, in which almost all studied fungal isolates of both the *C. haemulonii* species complex and *C. auris* exhibited high aggregation after 2 h of incubation at 37 °C. Interestingly, the fungal cells forming the aggregates remained viable. The aggregation of all isolates was not impacted by pH, temperature, β-mercaptoethanol (a protein-denaturing agent), or EDTA (a chelator agent). Conversely, proteinase K, trypsin, and sodium dodecyl sulfate (SDS) significantly diminished the fungal aggregation. Collectively, our results demonstrated that the aggregation ability of these opportunistic yeast pathogens is time-dependent, and surface proteins and hydrophobic interactions seem to mediate cell aggregation since the presence of proteases and anionic detergents affected the aggregation capability. However, further studies are necessary to better elucidate the molecular aspects of this intriguing phenomenon.

## 1. Introduction

The *Candida haemulonii* species complex is classically formed by the emergent pathogens *C. haemulonii sensu stricto*, *C. duobushaemulonii*, and *C. haemulonii* var. *vulnera* [1]. The globally concerning fungus *Candida auris* is phylogenetically associated with this fungal complex, and together they form the *C. haemulonii* clade [1]. Molecular methods are required for the correct identification of these emerging opportunistic yeasts [1]. Both the *C. haemulonii* species complex and *C. auris* are well-known for their intrinsic multidrug-resistance profiles to clinically available antifungal agents, particularly azoles and polyenes, which challenges clinicians to achieve successful treatments. This scenario is worsened by the medical condition of the affected individuals, who generally have a compromised immunological system and/or severe underlying illnesses such as diabetes, pulmonary, renal, and peripheral vascular diseases [2].

Although species belonging to the *C. haemulonii* complex have been reported in clinical cases around the world, they are still considered rare causative agents of human infections. However, some countries, such as India, Korea, Kuwait, and Brazil [2], have experienced an increase in the incidence of these fungi in recent years. The reasons for this circumstance are not clear. *Candida auris* is considered a serious global health threat by the Centers for Disease Control and Prevention (CDC, USA), mainly due to its capacity to cause outbreaks in healthcare settings. To our knowledge, the ability to cause outbreaks is the major difference between *C. auris* and the species comprising the *C. haemulonii* complex, which can be attributed to the ability of *C. auris* to resist the commonly used disinfectants, its persistence on medical devices and hospital surfaces for weeks, and its ability to colonize the axillae, nares, and groin of patients [3]. Both the *C. haemulonii* species complex and *C. auris* can cause superficial to deep-seated infections and produce a wide range of virulence attributes [2].

Cellular aggregation is a phenomenon that has been described in *C. auris*, and isolates can be classified as either aggregative or non-aggregative. However, the understanding of this aggregation phenotype is still being studied [4,5,6]. According to Borman and coworkers [4], aggregative strains of *C. auris* exhibit large aggregates (many cells attached to one another) that cannot be disrupted by vortex mixing or detergents. The aggregative phenotype has been associated with colonizing isolates, while the non-aggregative phenotype was mainly associated with isolates recovered from candidemia cases [5]. The comparison of virulence between aggregative and non-aggregative phenotypes is still controversial. In this sense, Borman and coworkers [4] demonstrated that aggregative *C. auris* isolates exhibited less virulence compared to non-aggregative ones in the *Galleria mellonella* larvae model. Conversely, *C. auris* aggregative isolates formed more robust biofilms than non-aggregative isolates [5]. On the other hand, Carvajal and coworkers [6] did not observe a clear relationship between the phenotype of aggregation and the virulence of *C. auris* isolates, including in vivo infection of *G. mellonella* larvae.

Brown and coworkers [7] demonstrated that aggregative isolates of *C. auris* were more cytotoxic than the non-aggregative ones using two- and three-dimensional skin epithelial models. Additionally, the genes *ALS5* and *SAP5*, associated with virulence traits and host invasion, were upregulated in the aggregative *C. auris*, which can be involved in the greater inflammatory response presented by this phenotype within the host tissue. *ALS5* is a key adhesion gene, and the upregulation of the adhesin protein Als5p in older cells of *C. auris* has been demonstrated, along with a thickened cell wall, decreased neutrophil killing, and increased epithelial adhesion [8].

The aggregative phenotype of *C. auris* has been proposed to be a consequence of a defect in cellular division and failure to release daughter cells after the budding event [4]. However, a recent study reported a new form of aggregation in *C. auris* resulting from increased adherence between adjacent cells, likely caused by genomic amplification of the cell wall adhesin *ALS4*, which plays a role in virulence in *G. mellonella* larvae as well as adhesion to both biotic and abiotic surfaces [9]. Regarding the *C. haemulonii* species complex, the aggregative phenotype has not been previously described, but our research group demonstrated that clinical isolates of the *C. haemulonii* complex exhibit a tendency to form aggregates [10,11]. However, very little is effectively known about this intriguing biological event in these emerging yeasts. Therefore, in this work, we aimed to study the aggregation phenomenon in clinical isolates belonging to the *C. haemulonii* species complex and *C. auris*, as well as the effects of both chemical and physical factors on their aggregation capability.

## 2. Materials and Methods

### 2.1. Microorganisms and Growth Conditions

A total of 18 clinical isolates of *C. haemulonii* species complex and *C. auris* were used in the present study. The isolates of the *C. haemulonii* complex were recovered from patients from Brazilian hospitals between 2005 and 2013 and were identified by molecular approaches as *C. haemulonii* (*n* = 5; LIP*Ch*2 recovered from the sole of the foot, LIP*Ch*3 from toe nail, LIP*Ch*4 from finger nail, LIP*Ch*7 from toe nail, and LIP*Ch*12 from blood), *C. duobushaemulonii* (*n* = 4; LIP*Ch*1 from finger nail, LIP*Ch*6 from toe nail, LIP*Ch*8 from blood, and LIP*Ch*10 from bronchoalveolar lavage), and *C. haemulonii* var. *vulnera* (*n* = 3; LIP*Ch*5 from toe nail, LIP*Ch*9 from urine, and LIP*Ch*11 from blood) [12]. *Candida auris* isolates were recovered from patients from Colombian hospitals between 2005 and 2016 (*n* = 6; Ca386 was recovered from a biopsy of bone tissue, Ca432 from the secretion of craniotomy, Ca485 from eye discharge, Ca446 and Ca885 from blood, and Ca881 from cerebrospinal fluid) [13]. Fungal cells were cultured in Sabouraud-dextrose broth (SDB) at 37 °C for 48 h and then used in all experiments. The yeast cells were quantified using a Neubauer chamber.

### 2.2. Aggregation Kinetic Assay

The aggregation assay was performed using a standard method previously described [14,15]. In this sense, yeasts grown in SDB were washed twice in sterile phosphate-buffered saline (PBS, pH 7.2), and then fungal suspensions containing 10^8^ yeasts/mL were prepared in microcentrifuge tubes (Eppendorf, Hamburg, Germany), vortexed for 30 s and then transferred by pipetting into plastic cuvettes (1 mL/cuvette). The fungal suspensions were incubated at 37 °C without agitation for 30-, 60-, 90-, and 120-min. Aggregation was quantified as a percentage reduction in the optical density (OD) and calculated as ([OD_0_ − OD_f_]/OD_0_) × 100, where OD_0_ is the OD value at the start of the experiment and OD_f_ is the value after the different incubation time periods. All measurements were performed at 530 nm using a spectrophotometer (Ultrospec 2100 Pro, Amersham Biosciences, Amersham, United Kingdom). PBS without fungal cells was used as a blank. The percentage of aggregation was calculated and used for classification of aggregation as follows: high (more than 40%), intermediate (30–40%), and low aggregation (less than 30%) [16]. One isolate of each *Candida* species was selected for the subsequent experiments.

### 2.3. Aggregation after Prolonged Periods and Assessment of Viability

The selected fungal isolates were prepared for aggregation assays as described above and incubated for 5 and 24 h at 37 °C. At each time point, the OD was read at 530 nm, and the percentage of aggregation was calculated as described above. In parallel, aliquots (10 μL) of cell suspensions were obtained at each time point, spotted on the surface of Sabouraud-dextrose agar (SDA) plates, and incubated at 37 °C for 48 h to evaluate the viability of the fungal isolates after aggregation. Fungal cells were also stained with a crystal violet solution (0.2% in water; Sigma-Aldrich, St. Louis, MO, USA) after each incubation period and observed using a light microscope to investigate cell viability. The dye is unable to enter viable (intact) cells, which remain unstained, while dead cells become blue due to the dye’s ingress. In this context, control of dead cells was obtained by boiling fungal cells for 20 min, followed by staining them with the same crystal violet solution.

### 2.4. Light Microscope Imaging

Fungi (10^8^ yeasts/mL) were incubated in PBS (pH 7.2) at 37 °C for 2 h to allow cell aggregation. Afterwards, the systems were gently mixed by pipetting, and an aliquot of 10 μL of each cell suspension was transferred to a glass slide, covered with a coverslip, and observed using a fluorescence microscope to obtain differential interference contrast images (DIC; Zeiss Fluorescence Microscope—Axio Imager D2 [Zeiss, Jena, Alemanha]). Aliquots (10 μL) of the cell suspensions taken before incubation were used as a control for negative aggregation for comparison purposes (time 0 h).

### 2.5. Effects of Chemicals on Aggregation

In order to investigate the influence of chemical factors on the aggregation ability of the clinical isolates of *C. haemulonii* species complex and *C. auris*, fungal suspensions were prepared as described above and then incubated for 2 h to allow aggregation in the following conditions: (i) PBS adjusted to pH 4.5, 7.2, and 8.5 to investigate the involvement of charged groups on aggregation capability [17]; (ii) 50 μg/mL of proteinase K (Invitrogen, Carlsbad, CA, USA); (iii) 0.25% trypsin (Nova Biotecnologia, São Paulo, Brazil) [9]; (iv) 0.05% to 0.25% sodium dodecyl sulfate (SDS) [18]; (v) 0.5% to 2% β-mercaptoethanol [18]; (vi) 0.5 mM to 5 mM ethylenediamine tetraacetic acid tetrasodium salt (EDTA; Sigma-Aldrich, St. Louis, MO, USA) [18]. DIC images were obtained as described above after incubation of the clinical isolates with the chemicals that affected their aggregation capability.

### 2.6. Effects of Temperature on Aggregation

To evaluate the influence of temperature on the aggregation capability of the isolates of the *C. haemulonii* species complex and *C. auris*, fungal cells were prepared as described above and then incubated for 2 h to allow aggregation at either 28 °C or 37 °C.

### 2.7. Statistics

All experiments were performed in triplicate, in three independent experimental sets. The results were analyzed statistically by Student’s *t*-test (in comparisons between two groups) and by the Analysis of Variance One-Way ANOVA followed by Dunnett’s multiple comparison test (in comparisons between three or more groups). All analyses were performed using the program GraphPad Prism 8. In all analyses, *p* values of 0.05 or less were considered statistically significant.

## 3. Results and Discussion

### 3.1. Aggregation Is a Time-Dependent Event in C. haemulonii Clade

**Figure 1:**
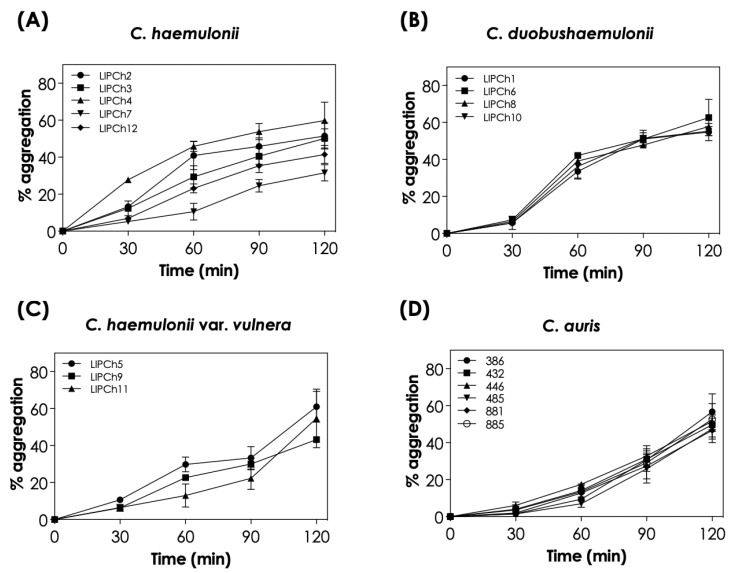
Aggregation capability of the clinical isolates comprising the *C. haemulonii* species complex and *C. auris*. The aggregation was evaluated by the reduction in the optical density (at 530 nm) of fungal suspensions in PBS (pH 7.2) containing the fungi (10^8^ yeasts/mL) after 30, 60, 90, and 120 min of incubation at 37 °C in inert conditions. The results were expressed as percentages of aggregation for each clinical isolate of *C. haemulonii* (**A**), *C. duobushaemulonii* (**B**), *C. haemulonii* var. *vulnera* (**C**), and *C. auris* (**D**). The values represent the mean ± standard deviation of three independent experiments.

The auto-aggregation capability of the 18 clinical isolates from the *C. haemulonii* species complex and *C. auris* studied herein was observed to be a time-dependent event (Figure 1). When analyzing each species individually, we observed that *C. auris* isolates had a very similar percentage of aggregation at each time point, with mean aggregation ranging from 3.1 ± 1.8% after 30 min to 50.8 ± 3.7% after 120 min of incubation. Similar results were observed for *C. duobushaemulonii* isolates, with the mean percentage of aggregation varying from 6.3 ± 0.8% after 30 min to 57.7 ± 3.6% after 120 min. On the other hand, *C. haemulonii* and *C. haemulonii* var. *vulnera* isolates exhibited a more variable profile among the different isolates. In this sense, the mean aggregation of *C. haemulonii* isolates varied from 13.1 ± 8.9% after 30 min to 46.9 ± 10.8% after 120 min, while the mean percentage of aggregation of *C. haemulonii* var. *vulnera* isolates was 7.9 ± 2.4% and 54.3 ± 11.4% after 30 and 120 min of incubation, respectively.

Supporting our observations, Tomici and coworkers [17] also demonstrated that the auto-aggregation percentage in isolates of *C. albicans*, *C. krusei*, *C. glabrata*, and *Saccharomyces boulardii* increases over time. Cellular aggregation is a well-known phenomenon in the microbial field, described in both bacteria and fungi, not only in natural environments but in mammalian hosts as well. For instance, nitrogen-fixing bacteria such as *Azospirillum*, *Klebsiella*, and *Azotobacter* can aggregate and flocculate, which contributes to their dispersal and survival in soil [18]. Microorganisms can exhibit the ability of auto-aggregation, characterized by the aggregation between cells of the same microbial strain, or coaggregation, characterized by the aggregation between different microbial strains or species, or even interkingdom interactions [19]. The formation of dental caries, for example, is highly mediated by the coaggregation process, and, for this reason, several coaggregation studies have focused on microorganisms of the human oral cavity, such as *Streptococcus salivarius* and *Candida albicans*, among others [19,20]. Additionally, the coaggregation of *Lactobacillus* with potential intestinal pathogens, such as *Escherichia coli* and *Klebsiella* spp., as well as some *Candida* species, as an anti-infection mechanism has also been investigated by research groups [17,21].

The global threat posed by *C. auris* depends in part on its described aggregative phenotype [4]. The impact of this phenotype on fungal cells and virulence is still being investigated, and studies are somehow contradictory, but it has been shown to influence biofilm formation, fungal virulence, and antifungal susceptibility, including tolerance to clinical concentrations of sodium hypochlorite, with some isolates persisting alive after 14 days of treatment [22].

Recently, Pelletier and coworkers [23] demonstrated that macrophages are unable to clear *C. auris* aggregates, which could benefit the fungi during a systemic infection by facilitating the persistence of infection. An elegant study conducted by Forgács and coworkers [24] showed the presence of large aggregates of *C. auris*, formed by single and budding yeast cells, in the kidneys, livers, and hearts of immunosuppressed mice after six days of infection. Moreover, despite exhibiting an aggregative or non-aggregative phenotype in vitro, the *C. auris* isolates presented the same behavior in vivo, and the authors speculate that these aggregates in tissues could protect the fungi from the host immune system [24].

All clinical isolates of the *C. haemulonii* species complex and *C. auris* that underwent testing demonstrated high aggregation after 2 h of incubation, according to the criteria established in this study (i.e., aggregation >40%), except for one *C. haemulonii* isolate (LIP*Ch*7), which showed intermediate aggregation. 

The following isolates were chosen for further experiments: LIP*Ch*4 from *C. haemulonii*, LIP*Ch*6 from *C. duobushaemulonii*, LIP*Ch*5 from *C. haemulonii* var. *vulnera*, and Ca386 from *C. auris*. *Candida duobushaemulonii* and *C. auris* isolates were randomly selected for the study because very little difference was observed in their aggregation capabilities after 2 h of incubation. In contrast, for *C. haemulonii* and *C. haemulonii* var. *vulnera*, the isolates that showed the highest aggregation ability were chosen for the further experiments. 

### 3.2. Aggregation after Prolonged Periods and Viability

**Figure 2:**
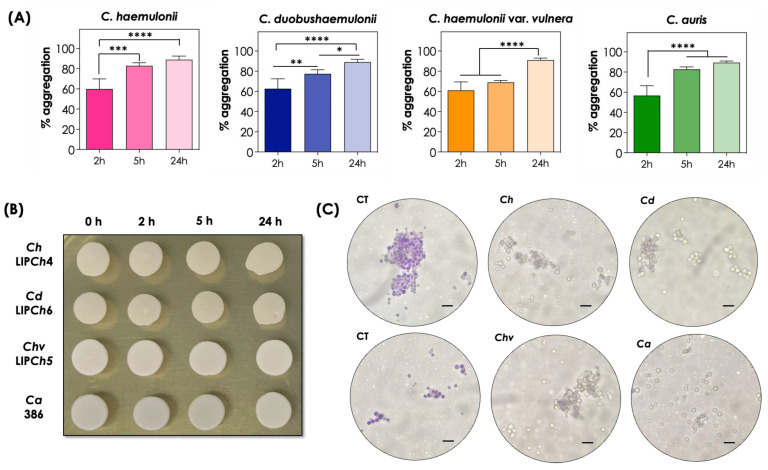
Aggregation capability and assessment of viability after long periods of time for the clinical isolates comprising the *C. haemulonii* species complex and *C. auris*. (**A**) The aggregation was evaluated by the reduction in the optical density (at 530 nm) of fungal suspensions in PBS (pH 7.2) containing the fungi (10^8^ yeasts/mL) after 0, 2, 5, and 24 h of incubation at 37 °C in inert conditions. The results were expressed as percentages of aggregation of isolates LIP*Ch*4 from *C. haemulonii*, LIP*Ch*6 from *C. duobushaemulonii*, LIP*Ch*5 from *C. haemulonii* var. *vulnera*, and Ca386 from *C. auris*. The values represent the mean ± standard deviation of three independent experiments. The asterisks mean the following: (****) *p* < 0.0001; (***) *p* < 0.001; (**) *p* < 0.01, and (*) *p* < 0.05. (**B**) Cellular viability was assessed by spot inoculation of 10 μL of each system on SDA after each time point. (**C**) Representative images of the viability of aggregates after 24 h of incubation, evaluated by staining with a 0.2% crystal violet solution. CT corresponds to the control of fungal cells boiled for 20 min prior to staining. *Ch*, *C. haemulonii*; *Cd*, *C. duobushaemulonii*; *Chv*, *C. haemulonii* var. *vulnera;* and *Ca*, *C. auris*. Bars = 30 μm.

The aggregation ability of the selected fungal isolates was evaluated for prolonged periods (5 and 24 h) of incubation in PBS under inert conditions. The results revealed that aggregation remained time-dependent and, except for the isolate LIP*Ch*5 of *C. haemulonii* var. *vulnera*, all the other isolates exhibited aggregation around 80% after 5 h and 90% after 24 h of incubation (Figure 2A). Similar results were observed with *C. albicans* and *C. krusei* ATCC strains, but not with *C. glabrata* isolates, which exhibited considerably lower aggregation ability after the same incubation periods [17].

Fungal cells remained viable after aggregation, as can be observed by the fungal growth on SDA after the different incubation periods (Figure 2B). Corroborating this finding, we stained the fungal cells with a crystal violet solution after the incubation periods and observed that the dye was unable to enter the cells, demonstrating their viability, including those forming the aggregates (Figure 2C). On the other hand, fungal cells boiled for 20 min (control of dead cells) and then stained with the same dye turned blue, indicating that the dye entered the dead cells (Figure 2C).

### 3.3. Morphological Analysis of Cellular Aggregation

**Figure 3:**
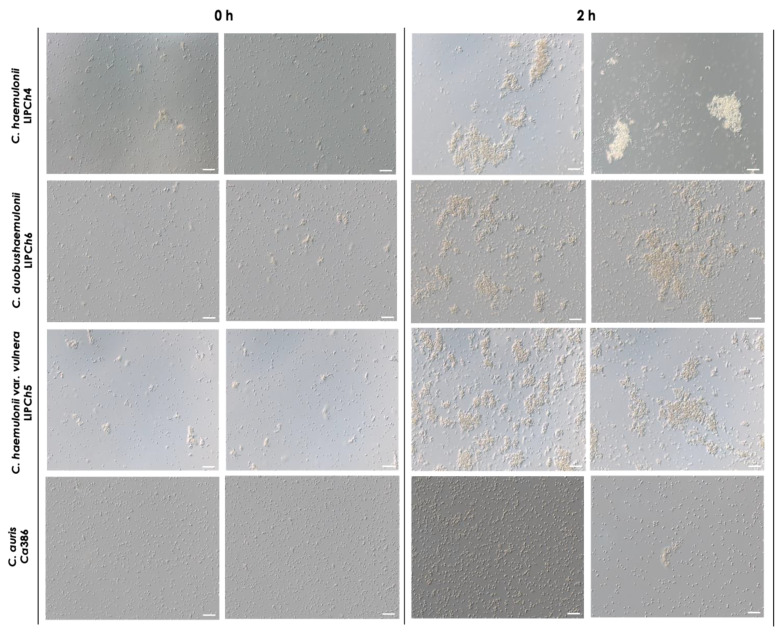
Aggregation of the clinical isolates comprising the *C. haemulonii* species complex and *C. auris*. The fungi (10^8^ yeasts/mL) were incubated at 37 °C for 2 h in PBS (pH 7.2), gently homogenized by pipetting, and observed using a light microscope. DIC images represent the isolates LIP*Ch*4 from *C. haemulonii*, LIP*Ch*6 from *C. duobushaemulonii*, LIP*Ch*5 from *C. haemulonii* var. *vulnera*, and Ca386 from *C. auris* before (0 h) and after incubation (2 h). Images were obtained at ×20 magnification. Bars = 50 μm.

The four clinical isolates belonging to the *C. haemulonii* clade selected in the present study were analyzed both before and after a 2-h incubation at 37 °C in PBS at pH 7.2. Interestingly, we observed that the members of the *C. haemulonii* species complex exhibited clusters of cells even after vigorous vortex mixing, which represents the time 0 h of the experiment. These aggregates became noticeably larger after the incubation period, indicating the occurrence of significant cell-to-cell interactions. In contrast, *C. auris* also displayed clusters of cells at 0 h, but these were considerably smaller than those observed in the *C. haemulonii* complex isolates and remained similarly sized after incubation. However, the number of aggregates and the number of cells per aggregate visibly increased, but these were clearly smaller in comparison with the *C. haemulonii* complex isolates (Figure 3).

Our clinical isolates could be seen as single and budding yeast cells before and after incubation, and the same was observed by other authors with aggregates of *C. auris* both in vitro [23] and in vivo, using an immunosuppressed murine model [24].

### 3.4. Effects of Chemical Factors on Aggregation

**Figure 4:**
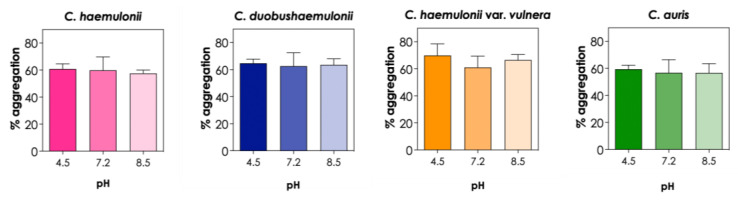
Effects of different pH values on the aggregation capability of clinical isolates of the *C. haemulonii* species complex and *C. auris*. The aggregation was evaluated by the reduction in the optical density (at 530 nm) of fungal suspensions in PBS (pH adjusted for 4.5, 7.2, and 8.5) containing the fungi (10^8^ yeasts/mL) after 2 h of incubation at 37 °C. The results were expressed as percentages of aggregation of isolates LIP*Ch*4 from *C. haemulonii*, LIP*Ch*6 from *C. duobushaemulonii*, LIP*Ch*5 from *C. haemulonii* var. *vulnera*, and Ca386 from *C. auris*. The values represent the mean ± standard deviation of three independent experiments.

The adaptation of opportunistic pathogens to different pH values favors their survival in the hostile environment of the human body, for example, facing basic pH in the mouth, acidic pH in the stomach, and neutral pH in the large intestine. In order to investigate the potential involvement of charged groups in the aggregation of *C. haemulonii* complex and *C. auris* isolates, we tested their ability to aggregate at different pH values. Since pH values ranging from 4.5 to 8.5 are relevant for biological systems, we evaluated the impact of PBS adjusted at three different pHs (4.5, 7.2, and 8.5) on the aggregation capability of the isolates and observed that none of the *Candida* species tested were affected by pH variation under the conditions used in our study (*p* > 0.05; Figure 4). On the other hand, Tomicic and coworkers [17] reported that the auto-aggregation of *C. albicans*, *C. krusei*, *C. glabrata*, and *S. boulardii* varied depending on pH values after 5 h of incubation, with the highest auto-aggregation observed at acidic pH (pH 4.5) and the lowest at basic pH (pH 8.5); however, after 24 h of incubation, no differences were observed in the auto-aggregation ability of these different *Candida* species.

**Figure 5:**
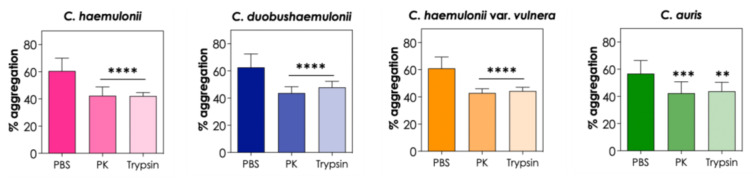
Effects of proteinase K (PK) and trypsin on the aggregation capability of the clinical isolates of the *C. haemulonii* species complex and *C. auris*. The aggregation was evaluated by the reduction in the optical density (at 530 nm) of fungal suspensions in PBS containing the fungi (10^8^ yeasts/mL), PK (50 μg/mL), and trypsin (0.25%) after 2 h of incubation at 37 °C. The results were expressed as percentages of aggregation of isolates LIP*Ch*4 from *C. haemulonii*, LIP*Ch*6 from *C. duobushaemulonii*, LIP*Ch*5 from *C. haemulonii* var. *vulnera*, and Ca386 from *C. auris*. The values represent the mean ± standard deviation of three independent experiments. The symbols represent the significant difference in aggregation capability between PBS and the PK or Trypsin systems. The asterisks mean the following: (****) *p* < 0.0001; (***) *p* < 0.001; (**) *p* < 0.01.

Subsequently, the fungi were treated with two broad-spectrum proteases, proteinase K and trypsin, to investigate whether surface proteins, such as adhesins, play a role in the aggregation process of *C. haemulonii* species complex and *C. auris*. Our results showed that both proteinase K and trypsin led to a significant reduction in the percentage of aggregation (*p* < 0.05), indicating that surface proteins are indeed important in cell-cell interactions that lead to aggregation in these emerging *Candida* species (Figure 5).

Bing and colleagues [7] demonstrated that treatment of *C. auris* with proteinase K and trypsin led to the separation of cell clumps into individual yeast cells in an isolate of *C. auris* that did not exhibit the typical aggregative phenotype; however, the enzymes were not able to disrupt the aggregates of a typical aggregative isolate of *C. auris*. Furthermore, quantitative transcriptional expression assays demonstrated that the relative expression levels of the *ALS4* gene in the typical aggregative isolate were comparable to those of a non-aggregative strain of *C. auris*, whereas the isolate whose aggregates were disrupted by the action of proteinase K and trypsin exhibited 400 times higher relative expression levels of the *ALS4* gene [9]. Therefore, those authors suggested the existence of two aggregative phenotypes in *C. auris*: the typical aggregative phenotype resulting from a defect in cell division and release of the budding daughter cells, and the new aggregative phenotype caused by the expansion of the *ALS4* gene adhesin [9]. Additionally, the authors demonstrated that the *C. auris* isolate with the new aggregative phenotype developed more robust biofilms on both polystyrene and silicone surfaces compared to the typical aggregative isolate and non-aggregative isolates of *C. auris*, which formed weaker biofilms [9]. Based on our findings, the clinical isolate of *C. auris* used in this study fits this newly described aggregative phenotype.

**Figure 6:**
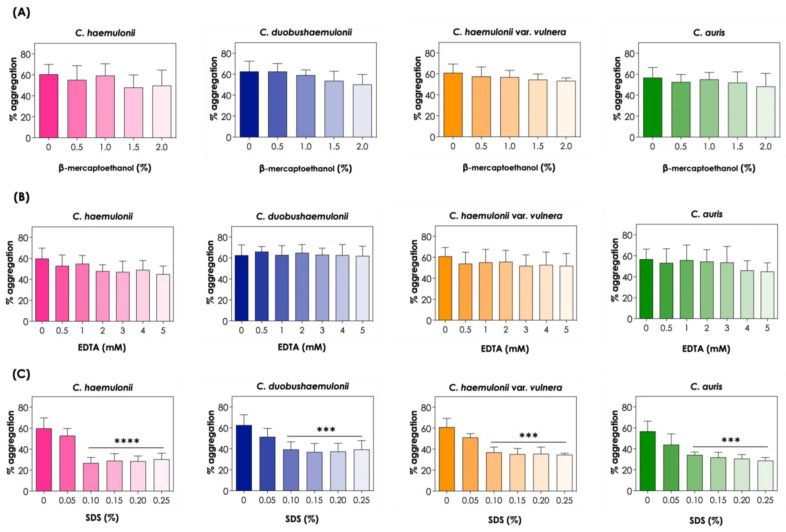
Effects of β-mercaptoethanol (**A**), EDTA (**B**), and SDS (**C**) on the aggregation capability of the clinical isolates of the *C. haemulonii* species complex and *C. auris*. The aggregation was evaluated by the reduction in the optical density (at 530 nm) of fungal suspensions (10^8^ yeasts/mL) after 2 h of incubation at 37 °C in PBS (pH 7.2) containing different concentrations of β-mercaptoethanol (0.5 to 2%), EDTA (0.5 to 5 mM), and SDS (0.05 to 0.25%). The results were expressed as percentages of aggregation of isolates LIP*Ch*4 from *C. haemulonii*, LIP*Ch*6 from *C. duobushaemulonii*, LIP*Ch*5 from *C. haemulonii* var. *vulnera*, and Ca386 from *C. auris*. The values represent the mean ± standard deviation of three independent experiments. The asterisks mean the following: (****) *p* < 0.0001; (***) *p* < 0.001.

In order to investigate the role of proteins in aggregation and other features related to cell adhesion, we utilized chemicals, such as the protein-denaturing agent β-mercaptoethanol, the chelator agent EDTA, and the anionic detergent SDS, to assess cell-to-cell interactions. In this sense, treatments with 0.5 to 2% β-mercaptoethanol and 0.5 to 5 mM EDTA had no effect on the aggregation ability of the clinical isolates tested, indicating that disulfide bonds and divalent cations did not appear to mediate aggregation in the *C. haemulonii* complex and *C. auris* (Figure 6A,B). Conversely, treatment with SDS significantly reduced the aggregation ability of all clinical isolates studied at concentrations varying from 0.10 to 0.25%, which suggests that hydrophobic interactions may play a role in cell-to-cell aggregation of isolates of the *C. haemulonii* complex and *C. auris* (Figure 6C).

It has been reported that the use of detergents is not capable of disrupting *C. auris* aggregates in isolates exhibiting the typical aggregative phenotype [4]. However, in this study, we demonstrated that SDS significantly reduced the aggregation of our isolate of *C. auris*, which did not present the typical aggregative phenotype, and the same effect was observed for the isolates of the *C. haemulonii* complex. To our knowledge, until now, no other studies have evaluated the impact of protein-denaturing and chelator agents on the aggregation ability of *Candida* spp. or other yeasts. Nevertheless, a study conducted with the bacterium *Azospirillum brasilense* also demonstrated that β-mercaptoethanol and EDTA did not affect bacterial aggregation [18].

**Figure 7:**
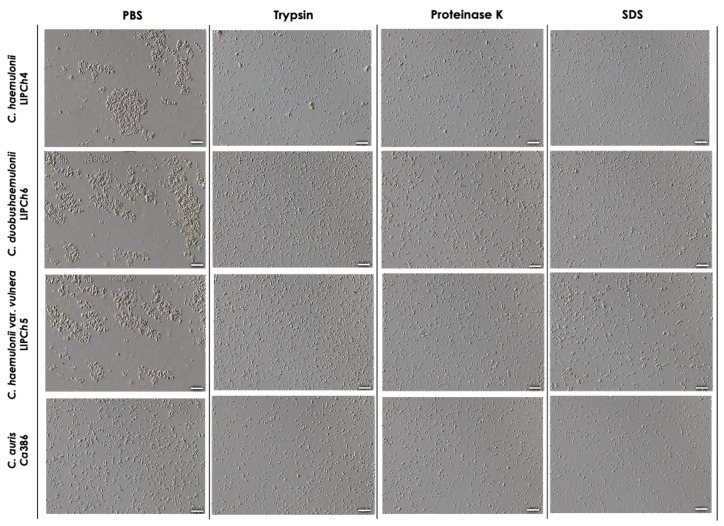
Effects of trypsin, proteinase K, and SDS on the aggregation capability of the clinical isolates belonging to the *C. haemulonii* species complex and *C. auris*. The fungi (10^8^ yeasts/mL) were incubated under inert conditions at 37 °C for 2 h in PBS (pH 7.2), trypsin (0.25%), proteinase K (50 μg/mL), and SDS (0.25%), gently homogenized by pipetting, and observed using a light microscope. DIC images represent the isolates LIP*Ch*4 from *C. haemulonii*, LIP*Ch*6 from *C. duobushaemulonii*, LIP*Ch*5 from *C. haemulonii* var. *vulnera*, and Ca386 from *C. auris* after incubation. Images were obtained at ×20 magnification. Bars = 50 μm.

Microscopic analyses confirmed the spectrometric results, revealing that the incubation of the clinical isolates of the four species tested herein with trypsin, proteinase K, and SDS drastically reduced their aggregation ability. As a result, the aggregates observed were considerably smaller than those observed in PBS systems (Figure 7).

### 3.5. Effects of Temperature on Aggregation

**Figure 8:**
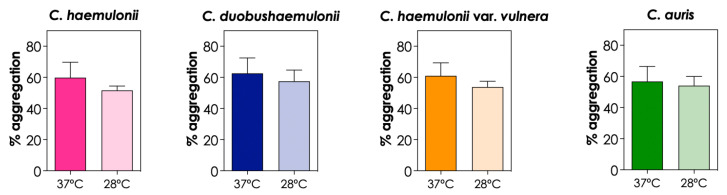
Effects of temperature on the aggregation capability of the clinical isolates of the *C. haemulonii* species complex and *C. auris*. The aggregation was evaluated by the reduction in the optical density (at 530 nm) of fungal suspensions (10^8^ yeasts/mL) after 2 h of incubation in PBS (pH 7.2) at 37 °C (human body temperature) and 28 °C (room temperature). The results were expressed as percentages of aggregation of isolates LIP*Ch*4 from *C. haemulonii*, LIP*Ch*6 from *C. duobushaemulonii*, LIP*Ch*5 from *C. haemulonii* var. *vulnera*, and Ca386 from *C. auris*. The values represent the mean ± standard deviation of three independent experiments.

All experiments were conducted at 37 °C to mimic the normal temperature of the human body. However, we also evaluated the aggregation ability of the isolates at room temperature (28 °C). The results indicated that the clinical isolates of *C. haemulonii* complex and *C. auris* tended to form fewer aggregates at 28 °C, but no significant differences were observed (*p* > 0.05; Figure 8). Tomicic and coworkers [9] reported that *C. krusei* and *C. glabrata* isolates exhibited a higher percentage of auto-aggregation at 37 °C compared to the same process at 28 °C and 42 °C; conversely, for *C. albicans*, a higher percentage of auto-aggregation was observed at 42 °C.

## 4. Conclusions

This study collectively demonstrated the ability of the *C. haemulonii* species complex and *C. auris* to form cell aggregates in a typically time-dependent manner. The presence of proteinase K, trypsin, and SDS significantly impacted the auto-aggregation process, suggesting that surface proteins and hydrophobic interactions play a crucial role in mediating the cell-to-cell adhesion of these *Candida* species. Nevertheless, further studies are necessary to better elucidate the molecular aspects of this intriguing phenomenon.

## Data Availability

Not applicable.
